# Toxic Constituents Index: A Toxicity-Calibrated Quantitative Evaluation Approach for the Precise Toxicity Prediction of the Hypertoxic Phytomedicine—Aconite

**DOI:** 10.3389/fphar.2016.00164

**Published:** 2016-06-17

**Authors:** Ding-kun Zhang, Rui-sheng Li, Xue Han, Chun-yu Li, Zhi-hao Zhao, Hai-zhu Zhang, Ming Yang, Jia-bo Wang, Xiao-he Xiao

**Affiliations:** ^1^China Military Institute of Chinese Medicine, 302 Military HospitalBeijing, China; ^2^College of Pharmacy, Chengdu University of Traditional Chinese MedicineChengdu, China; ^3^Research Center for Clinical and Translational Medicine, 302 Hospital of People’s Liberation ArmyBeijing, China; ^4^Key Laboratory of Modern Preparation of Traditional Chinese Medicine, JiangXi University of Traditional Chinese Medicine, NanchangChina; ^5^Integrative Medical Center, 302 Military HospitalBeijing, China

**Keywords:** toxic constituents index, aconite, toxicity prediction, multi-components determination, toxic potency, toxicity calibration coefficient

## Abstract

Complex chemical composition is an important reason for restricting herbal quality evaluation. Despite the multi-components determination method significantly promoted the progress of herbal quality evaluation, however, which mainly concerned the total amount of multiple components and ignored the activity variation between each one, and did not accurately reflect the biological activity of botanical medicines. In this manuscript, we proposed a toxicity calibrated contents determination method for hyper toxic aconite, called toxic constituents index (*TCI*). Initially, we determined the minimum lethal dose value of mesaconitine (MA), aconitine (AC), and hypaconitine (HA), and established the equation *TCI* = 100 × (0.3387 ×*X*_MA_ + 0.4778 ×*X*_AC_ + 0.1835 ×*X*_HA_). Then, 10 batches of aconite were selected and their evaluation results of toxic potency (*TP*), diester diterpenoid alkaloids (DDAs), and *TCI* were compared. Linear regression analysis result suggested that the relevance between *TCI* and *TP* was the highest and the correlation coefficient *R* was 0.954. Prediction error values study also indicated that the evaluation results of *TCI* was highly consistent with that of *TP*. Moreover, *TCI* and DDAs were both applied to evaluate 14 batches of aconite samples oriented different origins; from the different evaluation results, we found when the proportion of HA was reached 25% in DDAs, the pharmacopeia method could generate false positive results. All these results testified the accuracy and universality of *TCI* method. We believe that this study method is rather accurate, simple, and easy operation and it will be of great utility in studies of other foods and herbs.

## Introduction

The natural properties of botanical medicines determined the great variation of chemical composition and pharmacology activity (Li et al., 2007; Arceusz and Wesolowski, 2013; Ge et al., 2015). In order to evaluate the quality of herbal medicines, fingerprint identification and multi-components determination methods were widely applied as the main qualitative and quantitative methods recent years (Wu et al., 2011; Bian et al., 2013). Comparing with the single component assessment, these approaches are more effective. However, fingerprint technology could only assess the similarity of chromatograph outline, and could not associate with biological activity. Multi-components determination mostly concerned the total content of multiple components, and ignored the activity variation of each ingredient (Lin et al., 2014). In fact, there was no direct correlation between the activity and the content of chemical components. Some active components usually are the low abundance ones in medicinal plants, antitumor ingredient paclitaxel oriented from *Taxus cuspidata* is a good case (Zhang et al., 2015). Therefore, it is an interesting and challenge work to develop a quality evaluation method to reflect the activity and content of multiple components at the same time.

Aconite has played an important role in human history. In Indian, Korean, Japanese, and Chinese medicine systems, it has been used worldwide as poisons as well as an effective medicine. In the twentieth century, several reported toxicity cases directly decreased the use of aconite in herbal prescription in European and American countries (Nyirimigabo et al., 2015). Currently, the toxic ingredients and poisoning mechanism of aconite are well known. The strong cardiotoxicity and neurotoxicity mainly ascribed to the hyper toxic diester diterpenoid alkaloids (DDAs) and their actions on the voltage-sensitive sodium channels of the cell membranes in myocardium, nerves, and so on. Such DDAs, including aconitine (AC), mesaconitine (MA), and hypaconitine (HA), could increase the obvious changes of opening frequency and number of sodium channels, and finally induce arrhythmia, and even death (Manners et al., 1995; Fu et al., 2006). Until recent years, cases of poisoning related to taking aconite herbs or their preparation are reported in East Asian regions (Singhuber et al., 2009; Chan and Au, 2010; Chan, 2016). However, its particular potential in targeting chronic heart failure, rheumatism, pain, and various tumors has aroused the interests of clinicians again. For instance, in a recently completed randomized controlled trial, a traditional Chinese medicine containing aconite was proven to have higher efficacy for treating chronic heart failure than the current synthetic drug (Li et al., 2013). It is thus of great significance to ensure the safety of aconite products and related preparations (Chan, 2015).

Scientific evaluation standard is the key to ensure the safety application of aconite. Currently, a general method for assessing the safety is quantitative analysis the total of AC, MA, and HA by high performance liquid chromatography (HPLC), which was recommended in the Chinese Pharmacopoeia (Ch.P.) (Commission, 2015). However, the premise of adding contents directly is that the activity of various ingredients is nearly the same. If ignoring this premise, the results of multi-components determination may not accurately reflect the herbs quality, even generate converse result. Actually, the toxicity of HA is only as much as one-third of AC (Bisset, 1981; Nyirimigabo et al., 2015), which suggested that the pharmacopoeia method has certain irrationality. Another method for assessing the aconite toxicity is biological evaluation. This method is rather intuitive and reliable, which can assess the toxicity by observing the value of LD_50_, or the minimum lethal dose (MLD; Qin et al., 2012; Tong et al., 2013). However, it needs to waste a lot of animals, and is not suitable for large-scale detection of a numbers of samples (Qin et al., 2012; Kim et al., 2013).

In fact, this situation is rather common in natural medicine. Therefore, we proposed the efficacy calibrated contents determination method called effect constituents index (ECI). In order to figure out the ECI, we need to establish the multi-components determination methods and measure the biological or pharmacological activities of different constituents, respectively. For the constituents with the same mother nucleus structure and comparable activity, only to determine the activity in an appropriate concentration; while for those constituents with the different structure, it generally needs to determine the activity in a serials of concentrations and calculate the 50% effective concentration (EC_50_) values. According to these activity values, we can figure out the efficacy calibration coefficient for each ingredient, and further obtain the ECI equation. It should be noted that toxicity is one of the special form of efficacy, thus, we can calculate the toxic constituents index (*TCI*) using the same methods. The difference between ECI and *TCI* only was the type of evaluation index, the former mainly selected some active parameters, such as antibacterial, anti-inflammatory, cardiac, and so on, while the latter mainly used toxicity indicators, including the MLD, LD_50_, and cell proliferation inhibition rate. Three outstanding advantages for the ECI or *TCI* method should be mentioned. Initially, this method combined the advantages of chemical evaluation and biopotency assays, which makes it possible to represent the effect or toxicity of herbs by just determining the content of active constituents. Then, it solves the problem of the contribution difference between different ingredients, and the evaluation result was more close to the real activity of the sample. Last, this method did not increase the detection difficulty or cost of the existing chemical evaluation, once established the ECI or *TCI*, which was no need to consume animal or cell. This is more in line with the requirements of modern ethics.

**FIGURE 1:**
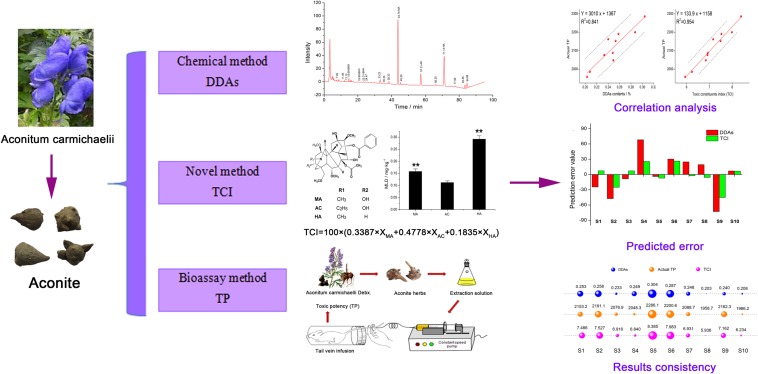
**The experimental flow chart**.

In the present study, we first proposed to study the *TCI* for the toxicity evaluation of aconite. Firstly, we determined the MLD value of MA, AC, and HA, and figured out their toxicity calibration coefficients. On this basis, we established the *TCI* method. In order to confirm the accuracy of *TCI*, 10 batches of aconite were chose, and the results of toxic potency (*TP*), DDAs, and *TCI* were compared. Last but not least, *TCI* and DDAs were both applied to evaluate aconite samples oriented from different origins; from the different evaluation results, we revealed the influence of components proportion on the evaluation results, and demonstrated the universality of *TCI*. **Figure 1** shows the experimental flow chart of this study. Our results will help to accurately evaluate and control the aconite toxicity; moreover, this research method can be used as a reference for other herbs and foods.

## Materials and Methods

### Ethics Statement

This study was conducted in strict accordance with the recommendations of the Guidelines for the Care and Use of Laboratory Animals of the Ministry of Science and Technology of China. The animal protocol was approved by the Committee on the Ethics of Animal Experiments of the 302 Military Hospital (Approval ID: IACUC-2013-054).

### Chemicals and Animals

Ten batches of crude aconite were harvested at their native cultivation site (Jiangyou, Sichuan Province, China) in early July. Another 14 batches of crude aconite were collected from Jiangyou, Hanzhong, Butuo, Weishan, and Anxian, respectively. Sample information was listed in **Table 1**. All the crude aconite samples, identified by Professor Xiao-he Xiao, were deposited at the China Military Institute of Chinese Materia Medica, 302 Military Hospital of China, Beijing, China.

**Table 1:** Raw herbs used in this work.

Sample number	Sources	Origins	Harvesting time	Description
S1–S10	Jiangyou, Sichuan	Daughter roots of *Aconitum carmichaelii* Debx.	June 2014	Fresh
JY1–JY3	Jiangyou, Sichuan	Daughter roots of *Aconitum carmichaelii* Debx.	June 2014	Fresh
HZ1–HZ3	Hanzhong, Shaanxi	Daughter roots of *Aconitum carmichaelii* Debx.	August 2014	Fresh
BT1–BT3	Butuo, Sichuan	Daughter roots of *Aconitum carmichaelii* Debx.	September 2014	Fresh
WS1–WS3	Weishan, Yunnan	Daughter roots of *Aconitum carmichaelii* Debx.	September 2014	Fresh
AX1–AX2	Anxian, Sichuan	Daughter roots of *Aconitum carmichaelii* Debx.	October 2014	Fresh

Standards of MA, AC, and HA were purchased from the National Institute for the Control of Pharmaceutical and Biological Products of China. The purity of the three standards was each above 98.0%, and the lot number was 111795-200901, 111794-200901, and 111796-201303, respectively.

HPLC grade acetonitrile and methanol were purchased from Fisher Chemicals (Pittsburg, PA, USA). The ultrapure water used in the experiments was prepared using a Milli-Q Ultrapure water purification system (Millipore, Bedford, MA, USA). Analytical grade dichloromethane, ethyl, isopropanol, concentrated ammonia, and hydrochloric acid were purchased from Beijing Chemical Reagents Company (Beijing, China). All the solutions were filtered through 0.22 μm membranes (Jinteng, Tianjin, China). Sodium chloride injection was get from Kelun Industry Group. WZ-502 micro-injection pump was supplied by Zhejiang University Medical Machinery Co., Ltd (Hangzhou, China).

Male Sprague-Dawley rats weighing 180–200 g were obtained from the laboratory animal center of The Military Medical Science Academy of the People’s Liberation Army (Permit No. SCXK-(A)2012-0004). The animals were maintained under controlled conditions of temperature 20 ± 0.5°C, humidity 55 ± 5% and with 12 h light and 12 h dark cycles. Before experiments, the animals fasted for 24 h with free access to water.

### Determination of DDAs Contents in Crude Aconite (Zhang et al., 2015)

Nowadays, there are many determination methods for DDAs by using HPLC. On the basis of Chen et al. (2010), we optimized the gradient elution conditions, and established an approach both for multi-components determination and fingerprint identification.

#### Apparatus and Conditions of HPLC

The HPLC analysis was performed on an Agilent 1200 HPLC^TM^ System equipped with a quaternary solvent delivery pump, auto sampler, and UV detector connected to Agilent Chemstation software. The chromatographic separation was performed using a Phenomenex Gemini C_18_ column (250 × 4.6 mm, 5 μm) at 30°C. The mobile phase consisted of (A) acetonitrile—40 mM ammonium acetate in water at a pH of 10 adjusted with aqua ammoniae (25:75, v/v) and (B) acetonitrile—40 mM ammonium acetate in water at a pH of 10 adjusted with aqua ammoniae (65:35, v/v) using a gradient program of 10–22% B for 0–20 min, 22–37% B for 20–30 min, 37–47.5% B for 30–40 min, 47.5–52% B for 40–45 min, 52–60% B for 45–65 min, 60–75% B for 65–75 min, and 75–95% B for 75–80 min with a mobile flow rate of 0.8 ml/min. The detection wavelength was set to 235 nm for a sample injection volume of 10 μl.

#### Preparation of the HPLC Standard and Sample Solutions

A mixed standard solution containing 101.6 μg/ml MA, 49.2 μg/ml AC, and 100.0 μg/ml HA was prepared by adding an accurately weighed amount of each standard stock into volumetric flasks and dissolving them with MeOH-HCl (100:0.05, v/v). These solutions were stored in dark glass bottles at 4°C and were stable for at least 1 week. Working standard solutions were freshly prepared by diluting the appropriate amounts of the above solutions with MeOH-HCl (100:0.05, v/v) before injection.

To avoid changes during the drying process, the crude aconite was washed and cut into thin slices and dried using a freezer dryer. All the aconite samples were ground into a fine powder before extraction. A total of 2 g of the powder was accurately weighed and extracted with 3 ml of ammonia solution and 50 ml of a mixed solution of isopropanol-ethyl acetate (1:1) via ultrasonic extraction for 30 min. The extracted solution was cooled, which contributed to weight loss during the extraction procedure, and filtered through qualitative filter paper to yield the filtrate. We then measured 20 ml of the filtrate and placed it in a stink cupboard until the solution was recovered. We used 3 ml of a mixed solution of isopropanol–dichloromethane (1:1) to dissolve the residue, and then filtered it through 0.22 μm micropore film to yield the sample solution for HPLC.

### Determination of the *TP* for Crude Aconite (Qin et al., 2012)

Aconite is a hyper-toxic substance, and its alcohol extract can easily kill rats via tail vein injection. The MLD was recorded, and further calculated *TP* value. The bigger the *TP*, the stronger the real toxicity.

#### Standards

The AC standard was accurately weighed and dissolved in absolute ethanol to produce a 10 μg.ml^–1^ standard solution on the day of the assay.

#### Sample Extraction

To avoid changes during the drying process, all crude aconite samples were washed, cut into thin slices and then dried using a freezer dryer. Then, all were ground into a fine powder before extraction. A total of 2 g of the powder was accurately weighed and extracted with 20 ml of 70% ethanol via ultrasonic extraction for 30 min. After centrifugal treatment with the conditions of 5000 rpm for 10 min, the supernatant was separated. We measured 0.5 ml of supernatant and diluted it with 9.5 ml of normal saline; then, we filtered it through a 0.22 μm micropore film to yield the test solution.

#### Assay

When measured, each animal was fastened and a fine needle connected to a micro burette was inserted into its vein. The standard solution or test solution was slowly administered via intravenous infusion, ensuring the rapid onset of drug action, until the animal was dead. Some symptoms can be considered as the critical point of death, such as the dilation of pupil and cessation of breath. Six animals each were used for the test group and the standard group. The infusion volume and body weight were recorded, and the MLD was figured out.

#### Statistical Analysis

Based on the direct determination method in bioassay, the *TP* of 1 mg AC was identified as 1000 U. After the MLD results of standards solution and test solution were substituted into the Ch.P. bioassay statistical procedures BS2000 software, we can calculate the *TP* for each sample.

### Establishment of *TCI*

#### Determination of the MLD for DDAs

According to the determination method in Section “Determination of the TP for Crude Aconite”, the MLD of AC, MA, and HA were determined to assess their toxicity differences.

Each standard was accurately weighed and dissolved in absolute ethanol to produce a 200 μg.ml^–1^ standard solution, and then diluted it to generate a 10 μg.ml^–1^ standard solution using sodium chloride injection before tail vein injection. Each standard solution was slowly administered by intravenous infusion and the injection volume and body weight were recorded to figure out MLD value. Six animals used for each group as well as the below crude aconite samples.

Data were expressed as means ± standard deviation. One-way analysis of variance was used to compare the difference between groups. *P*-values <0.05 were considered statistically significant. All statistical analysis was performed using SPSS software version 22.0 (SPSS Inc., Chicago, IL, USA).

#### Calculation of *TCI* (Xiong et al., 2014)

It is known that the greater the MLD value, the smaller the virulence. Thus, we figured out the toxicity calibration coefficient for each ingredient using the normalization reciprocal value of MLD. The coefficients computing formula was listed in Eq. 1, and *TCI* computing formula was listed in Eq. 2.

(1) $$\begin{array}{c} W_{i}=\frac{1/MLDi}{\Sigma_{i=1}^{n}1/MLDi} \end{array}$$

(2) $$\begin{array}{c} TCI=100\times \overset{n}{\underset{i=1}{\Sigma }}Wi\times Xi \end{array}$$

In the Eqs 1 and 2, *W*_i_ is the toxicity calibration coefficient for each ingredient, *X*_i_ is the determined content, *n* is the number of toxicity ingredients.

### Comparison of Accuracy and Reliability between *TCI* and DDAs

*TP* represented the actual toxicity of samples. In order to test the accuracy of *TCI* evaluation results, 10 batches of aconite (S1–S10, listed in **Table 1**) were chose as research objects, and the results of DDAs, *TCI*, and *TP* were compared.

#### Determination of *TP*, DDAs, and *TCI*

Determination methods of DDAs, *TP*, and *TCI* referred to Sections “Determination of DDAs Contents in Crude Aconite,” “Determination of the *TP* for Crude Aconite,” and “Establishment of *TCI*,” respectively.

#### Correlation Analysis (Dawidowicz and Wianowska, 2009)

Horizontal axis shows the DDAs contents or *TCI* results, and the vertical axis shows the *TP* results. The simple correlation analysis was adopted to test the similarity and correlation coefficients between DDAs and *TP*, *TCI*, and *TP* using SPSS 22.0 software, respectively, and finally figured out regression equation and the correlation coefficient *R*. The larger the *R* value, the more accurate the method.

#### Prediction Error (Tung et al., 2015)

Based on the regression equation obtained above, we could calculate the predicted *TP* of each sample. Using the actual *TP* and predicted *TP*, we could get the prediction error value, and the calculation equation was listed in Eq. 3.

(3) $$\begin{array}{cc} \text{Prediction error value = Predicted TP value }-\;Actual\;TP\;value & \end{array}$$

In this equation, actual *TP* is the measured value according to Section “Determination of *TP*, DDAs, and *TCI*,” while predicted *TP* is the calculated results according to the regression equation obtained in Section “Correlation Analysis.”

#### Prediction Results Comparison of Mass Samples

Based on the prediction results obtained from Section “Prediction Error,” we compared the actual *TP* results and prediction *TP* results in 10 batches of aconite in the form of bubble area, created by the origin 9.2 software. The larger the bubble area, the stronger the toxicity. Through the bubble size and the arrangement order in the group, we can directly evaluate the prediction ability of *TCI* and DDAs methods for mass samples.

### Application of *TCI*

In order to investigate the application of *TCI*, another 14 batches of aconite from different origins (JY1–AX2, listed in **Table 1**) were chose. *TCI* and DDAs were used to assess these samples respectively, and their evaluation results were compared. For those samples had diversity evaluation results, we utilized multivariate statistical method to analyze the reason.

#### Determination of DDAs, and *TCI*

Determination methods of DDAs and *TCI* referred to Sections “Determination of DDAs Contents in Crude Aconite” and “Establishment of *TCI*,” respectively, and the evaluation results of two methods were compared.

#### Partial Least Squares-Discriminate Analysis (Zhang et al., 2012)

All samples were classified into two groups. Those ones had the same evaluation results in two methods were divided into one group, while those had different results were divided into another group. In order to reveal the reason, such multivariate analyses were utilized. The data from each sample were introduced to the software SIMCA-P 11.0 (Umetrics, Umea, Sweden) where partial least squares-discriminate analysis (PLS-DA) was used for calculation.

## Results

### DDAs Test

**FIGURE 2:**
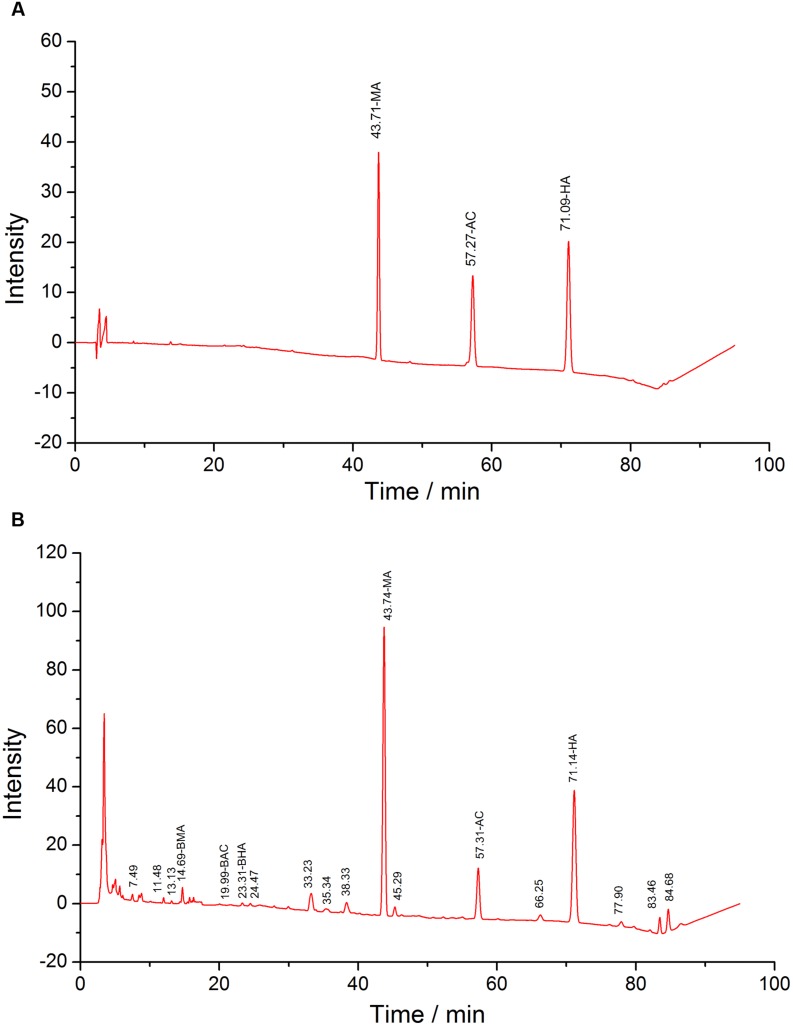
**HPLC of standards (A) and test solutions (B), the retention time of MA, AC, and HA are 43, 57, and 71 min, respectively**.

DDAs results of ten batches were illustrated in **Table 2** and the HPLC profiles were listed in **Figure 2**. It could be found that the DDAs content in S5 was the highest, while in S8 was the lowest. However, it also could be discovered that the results of *TP* and DDAs were not entirely consistent. For instance, the *TP* of S3 was higher than that of S4, but the DDAs in S3 was slightly lower than S4. This may be closely related to the real toxicity difference of several ingredients and their proportion difference in samples. As we known, AC is the most famous ingredient in aconite herbs; however, its proportion in DDAs was the least, and only about 10%. AC contents difference is not obvious between different batches. The rest contents are dominated by MA and HA. Therefore, aconite real toxicity primarily depends on the toxicity strength and proportions of MA and HA.

**Table 2:** Determined results of DDAs contents, and *TCI* of 10 batches aconite.

Batch	MA (%)	AC (%)	HA (%)	MA+AC (%)	HA+AC (%)	MA+HA (%)	DDAs (%)	*TCI*
1	0.151	0.017	0.085	0.168	0.102	0.236	0.253	7.486
2	0.142	0.02	0.096	0.162	0.116	0.238	0.258	7.527
3	0.136	0.018	0.079	0.154	0.097	0.215	0.233	6.916
4	0.116	0.016	0.117	0.132	0.133	0.233	0.249	6.840
5	0.141	0.021	0.142	0.162	0.163	0.283	0.304	8.385
6	0.139	0.019	0.129	0.158	0.148	0.268	0.287	7.983
7	0.123	0.016	0.109	0.139	0.125	0.232	0.248	6.931
8	0.114	0.015	0.074	0.129	0.089	0.188	0.203	5.936
9	0.136	0.022	0.082	0.158	0.104	0.218	0.240	7.162
10	0.114	0.022	0.072	0.136	0.094	0.186	0.208	6.234

### *TP* Test

*TP* results of 10 batches were listed in **Table 3**. All rats were dead within several minutes, with tremors or stool evacuation observed before death. According to the experimental results, AC is a hyper toxic substance, and an injection of 1.2–1.6 ml of 10 μg.ml^–1^ standard solution injection can kill rats easily via the tail vein. Meanwhile, other test samples extracted from 10 batches crude aconite also have very strong lethal toxicity. However, the toxicity in different batches aconite was not consistent in spite of all samples collected in the same producing region. To be more precise, aconite in S5 had the strongest toxicity, followed S6, S2, and S1. It seemed that S8 and S10 have the lowest one.

**Table 3:** Determined results of MLD and actual TP of 10 batches aconite.

Exp no.	DS-aconitine	DT-S1	DT-S2	DT-S3	DT-S4	DT-S5	DT-S6	DT-S7	DT-S8	DT-S9	DT-S10
1	73.46	63.39	69.36	61.37	63.53	57.52	71.44	60.51	67.69	55.41	78.39
2	59.70	71.97	67.39	70.97	66.35	64.7	56.19	63.29	76.38	59.72	71.53
3	77.67	70.13	68.47	62.55	72.45	53.62	62.3	61.54	71.69	68.54	67.48
4	66.96	58.55	57.57	72.39	74.35	60.44	58.62	71.53	65.37	62.28	62.41
5	65.22	59.09	59.55	61.58	60.32	57.1	61.04	67.65	75.71	66.43	70.54
6	69.14	59.67	54.22	67.53	65.08	66.96	64.77	69.51	63.55	68.63	64.37
TP/g		2153.2	2191.1	2076.9	2048.3	2286.1	2200.6	2088.7	1958.7	2162.3	1986.2

*DS, minimum lethal dose for standard sample; DT, minimum lethal dose for test sample.*

### Establishment of *TCI* and the Test

**FIGURE 3:**
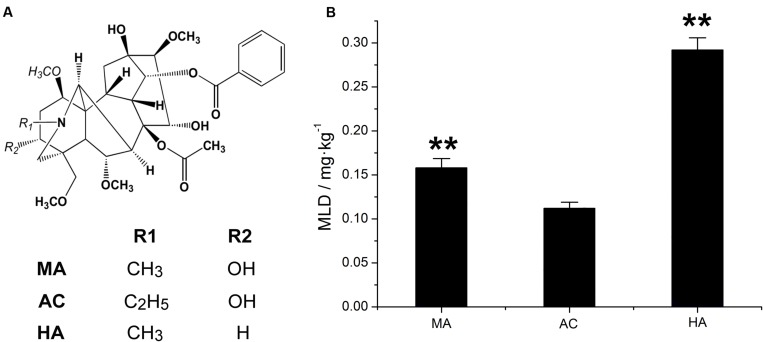
**Chemical structure of MA, AC, and HA (A), and their minimum lethal dose (MLD) value via tail intravenous injection in rats, ^∗∗^*P* < 0.01, vs aconitine standard group (B)**.

The chemical structure and MLD values of MA, AC, and HA were showed in **Figure 3**. It was found that the average MLD of MA, AC, and HA were 0.1580, 0.1121, and 0.2919 mg.kg^–1^, respectively. Among three kinds of DDAs, AC was the most poisonous substance, followed by MA and HA. It should be mentioned that the toxicity of HA only equaled to about one-third of AC.

According to the normalization reciprocal value method, the toxicity calibration coefficient for MA, AC, HA was 0.3387, 0.4778, and 0.1835, respectively. Therefore, the *TCI* equation was as follows:

(4) $$\begin{array}{c} TCI=100\times \left(0.3387\times X_{\text{MA}}+0.4778\times X_{\text{AC}}+0.1835\times X_{\text{HA}}\right) \end{array}$$

In this equation, *X*_MA_, *X*_AC_, and *X*_HA_ are the determined content of MA, AC, and HA, respectively.

Based on the Eq. 4, we calculated the **TCI** of 10 batches of aconite. The result was also illustrated in **Table 2**. It was clear that the *TCI* in S5 and S6 were the highest, while that in S8 and S10 were the lowest ones.

### Correlation Analysis Test

**FIGURE 4:**
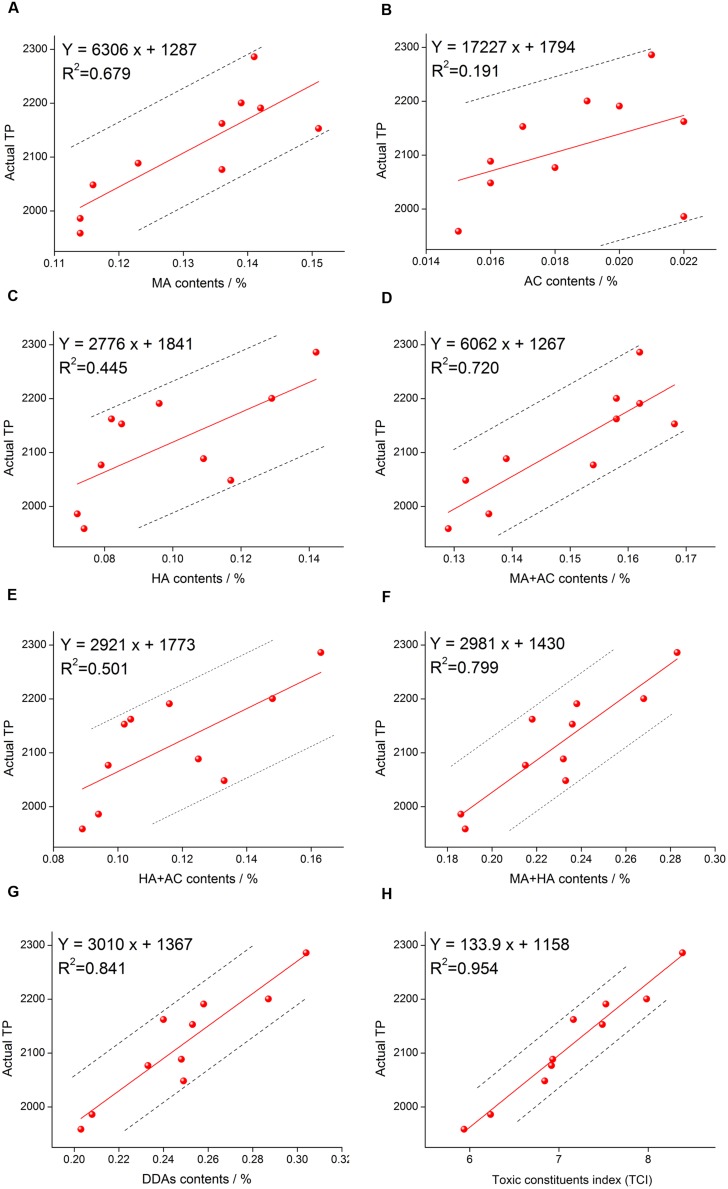
**Correlation analysis results of MA and *TP* (A), AC and *TP* (B), HA and *TP* (C), MA+AC and *TP* (D), HA+AC and *TP* (E), MA+HA and *TP* (F), DDAs (MA+AC+HA) and *TP* (G), and *TCI* and *TP* (H)**.

In order to judge the accuracy of different methods, we analyzed the correlation between DDAs, *TCI*, and *TP*. Analytical method was simple linear correlation and carried out using Excel 2007 software. The correlation analysis results were showed in **Figure 4**. According to the *R* value, it was clear that the correlation between single alkaloid content and actual *TP* was rather low, the highest one was MA and the *R* was 0.679, while the lowest one was AC and the *R* was only 0.191. With the increase of the inclusion components, the correlation increased gradually. When the three components are included, the correlation reached 0.841. The evaluation accuracy of the total amount of three components was significantly better than one or two components. To some extent, multi-components determination method for herb quality evaluation is better than single component determination. Moreover, when the content of the three components was converted to *TCI*, we discovered that the correlation between *TCI* and actual *TP* reached 0.954, which was significantly higher than that of DDAs. These results suggested that *TCI* is more accurate than DDAs for the evaluation of aconite toxicity.

### Prediction Results Comparison of Single Sample and Mass Samples

**FIGURE 5:**
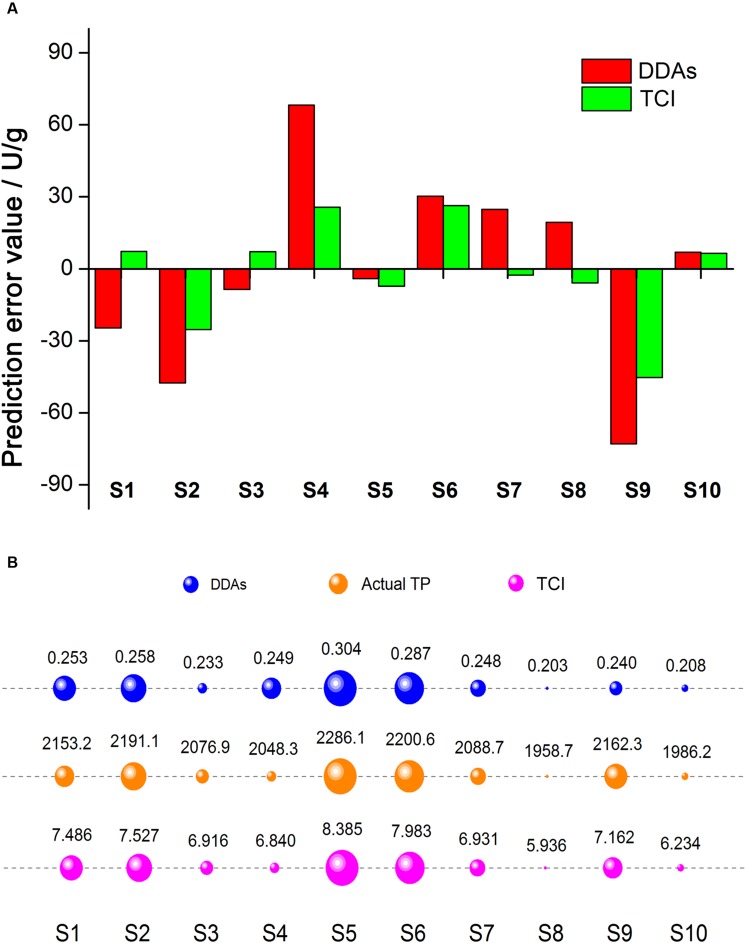
**Prediction error value comparison of DDAs and *TCI* (A).** Red and green represented DDAs and *TCI*, respectively. Prediction error value = Predicted *TP* value – Actual *TP* value. The evaluation results comparison of DDAs, *TCI*, and actual *TP* **(B)**. Blue, orange, and magenta represented DDAs, actual *TP*, and *TCI*, respectively. The larger the bubble area, the stronger the toxicity.

To compare the evaluation results accuracy for single sample, we figured out the prediction error value using the fitted linear equation *Y* = 3010 *x* + 1367, and *Y* = 133.9 *x* + 1158, respectively, and the results were illustrated in **Figure 5A**. Focusing on the prediction error value, most sample error values obtained from *TCI* were less than that predicted by DDAs, while the only exception occurred in S5, and the prediction error from *TCI* was slightly higher than that of DDAs. This could be understood that *TCI* was beneficial to reduce the prediction error for most samples when the measurement of aconite toxicity.

For further assessment of the evaluation results accuracy for mass samples, we compared the consistency of three methods (**Figure 5B**), where the actual *TP* results was as the reference. The area of bubble represented the toxicity, the bigger the bubble, the stronger the toxicity. From the order of the bubble, we observed that the evaluation results of DDAs were significantly different from that of *TCI*, and the controversial samples distributed in S1, S3, S4, and S9. It is remarkable that the DDAs contents of S1 was stronger than S9, while the actual *TP* in S1 was much lower, the same situation also appeared in S3 and S4. When converting DDAs into *TCI*, we noticed the increase of evaluation results consistency. In addition to S1 and S9, the order of toxicity strength in other samples was consistent in both *TCI* and actual *TP*. These results showed *TCI* method could improve the evaluation accuracy of mass samples.

### *TCI* Used for the Toxicity Evaluation of Crude Aconite from Different Origins

**FIGURE 6:**
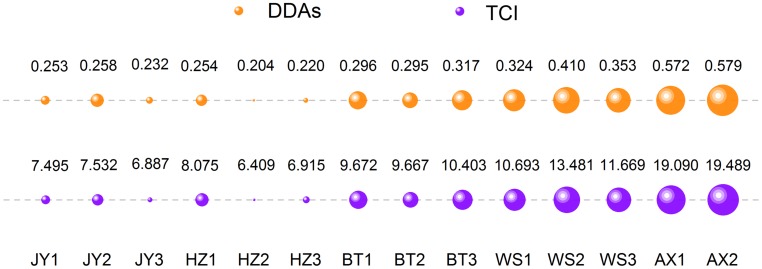
**Evaluation results comparison of DDAs and *TCI*.** Orange and purple represented DDAs, and *TCI*, respectively. The larger bubble area, the stronger toxicity.

By using *TCI* and DDAs methods, an accurate assessment for the toxicity from different origins was carried out and the results were demonstrated in **Table 4**. It was clear that the toxicity of crude aconite from different producing areas were different from each other no matter in the view of DDAs or *TCI*. Globally, the toxicity of Anxian aconite was the strongest, followed Weishan aconite, while the Jiangyou aconite and Hanzhong aconite from their native areas were the weakest ones. However, after a careful comparison for the toxicity of 14 batches of aconite, we could find an interesting phenomenon (**Figure 6**). In the DDAs view, the order of toxicity strength was HZ-2, HZ-3, JY-3, JY-1, HZ-1, JY-2, BT-2, BT-1, BT-3, WS-1, WS-3, WS-2, AX-1, and AX-2, while in the *TCI* view, the order was HZ-2, JY-3, HZ-3, JY-1, JY-2, HZ-1, BT-2, BT-1, BT-3, WS-1, WS-3, WS-2, AX-1, and AX-2. Those aconite could be classified into two groups according to the results consistency of two methods. Basically, samples with diverse results could be divided into group I, including JY-1, JY-2, JY-3, HZ-1, HZ-2, and HZ-3; while samples with consistent results could be divided into group II, including BT-1, BT-2, BT-3, WS-1, WS-2, WS-3, AX-1, and AX-2. This difference might be explained by the proportion variation of three alkaloids.

**Table 4:** Determined results of DDAs contents, and *TCI* of 14 batches aconite.

Batch	MA (%)	AC (%)	HA (%)	DDAs (%)	*TCI*
JY-1	0.151	0.0171	0.0852	0.253	7.495
JY-2	0.142	0.0203	0.0955	0.258	7.532
JY-3	0.136	0.0175	0.0787	0.232	6.887
HZ-1	0.150	0.0371	0.0666	0.254	8.075
HZ-2	0.132	0.0209	0.0512	0.204	6.409
HZ-3	0.135	0.0264	0.0589	0.220	6.915
BT-1	0.223	0.0268	0.0457	0.296	9.672
BT-2	0.225	0.0259	0.0441	0.295	9.667
BT-3	0.239	0.0299	0.0479	0.317	10.403
WS-1	0.257	0.0256	0.0417	0.324	10.693
WS-2	0.319	0.0342	0.0568	0.410	13.481
WS-3	0.280	0.0285	0.0449	0.353	11.669
AX-1	0.437	0.0613	0.0741	0.572	19.090
AX-2	0.446	0.0660	0.0670	0.579	19.489

**FIGURE 7:**
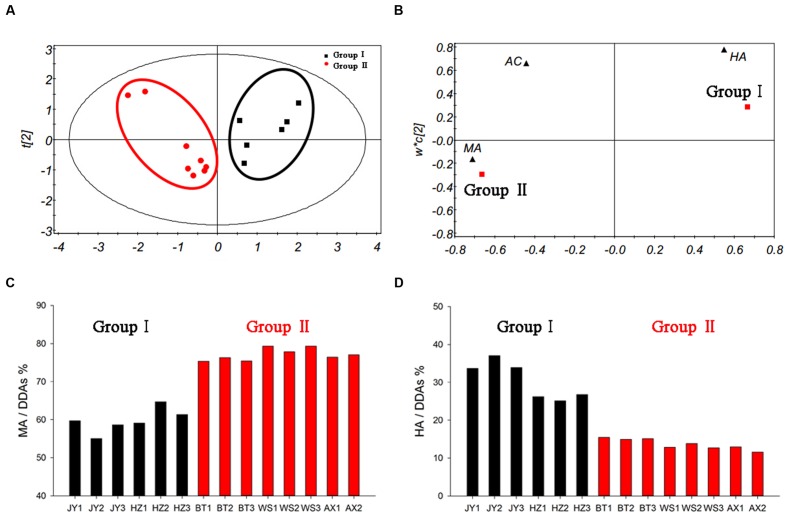
**PLS-DA model of crude aconite from different origins (A), loading plot (B), the proportion of MA in DDAs (C), and the proportion of HA in DDAs (D)**.

In order to explore the effects of components proportion on the toxicity, we utilized PLS-DA analysis to study it. The established PLS-DA model could describe 95.7% of the variation in *X* (*R*^2^*X* = 0.957) and 80.9% of the variation in the response *Y* (class) (*R*^2^*Y* = 0.809) with a predictive ability of 76.3% (*Q*^2^*Y* = 0.763). The results showed that a well-fitting PLS-DA model had been established. As illustrated in **Figure 7A**, the score plot showed that samples with diverse results and those with consistent results could be classified clearly. Meanwhile, the loading plot (**Figure 7B**) showed the relationship between variables and observations. It was clear that the classification of group I was dominated by HA, while the group II was dominated by MA. These results confirmed the fact that the toxicity assessment results were decided by the contents of MA and HA.

Further analysis for the proportion of MA and HA in these samples, some interesting rule was found (**Figures 7C,D**). The ratio of low-toxic substance HA in group I was between 25 and 40%, while in group II was less than 16%; on the contrary, the proportion of high-toxic ingredient MA in group I was between 55 and 65%, while in group II was higher than 75%. It is worth mentioning that the ratio of AC in DDAs was relatively fixed in all samples, which was approximately 10%. Thus, significant differences in the proportion of MA and HA were the main reason resulting in different evaluation results in DDAs and *TCI* method. It also suggested that when the proportion of HA was higher than 25% in samples, there was possible risk for incorrect judgment using DDAs method. After toxicity calibration and converted DDAs into *TCI*, we could overcome this deficiency, which also proved the universality of the *TCI* application.

### DISCUSSION

### Technical Advantages of *TCI*

The efficacy of natural medicines is the comprehensive action results of a variety of components, and the features of multi-component and multi-target have been confirmed (Tian and Liu, 2012; Wang et al., 2012; Zhang et al., 2014). Due to different chemical structures, the activity or toxicity of different components are not entirely consistent, and even great variation (Xu et al., 2014). For instance, AC is a hypertoxic substance in aconite, when the ester bond at the eighth position removed after heating and turns into benzoylaconitine, the toxic is less than 1/200 of the former (Qin et al., 2012). In addition, natural medicines from different producing areas, usually accompany significant variation in the content of some components (Song et al., 2014). Some scholars even suggested dividing the chemotype of plants based on this variation. Many plants have a variety of chemotype, such as *Litsea cubeba*, *Cannabis sativa* L., *Lippia origanoides* kunth, and so on (Abdul Hammid and Ahmad, 2015; Peschel and Politi, 2015; Sarrazin et al., 2015). In addition, for some drugs or foods, their chemical evaluation results and biological ones are not very consistent (Kaškonienė et al., 2015; Low et al., 2015), which also indirectly supported our understanding. Therefore, we proposed the active correction determination method. Our experiment also found that *TCI* method is more accurate than DDAs.

Biological evaluation results are certainly accurate, but there are some drawbacks. Compared with chemical testing, technical requirements of bioassay are relatively higher, and some experiments need large precision instruments, which results in not all organizations have the ability to carry out. In addition, other deficiencies limit the application of bioassay, including a large number of animals waste, high testing costs and low detection rate. However, *TCI* overcomes the shortcomings. Once established the equation, we need not to carry out the bioassay. So we can calculate the herb quality as long as bringing contents determination results into the equation. This will not increase the cost of testing, nor animal waste, but also improve the accuracy.

### Deficiencies Analysis

There are some deficiencies in this manuscript. Initially, we only took three main hypertoxic ingredients into *TCI* equation. Actually, other toxic components, such as indaconitine, beiwutine, also needed to be brought into equation in future work. Then, this method is not only suitable for the quality evaluation of aconite herbs, but also appropriate for the aconite products after detoxication processing. However, this manuscript did not introduce the results of aconite products due to some technical challenges in verification test. As we known, the toxicity of aconite significantly decreased after detoxification treatment. At this moment, it is extremely difficult to kill a rat and determine the MLD value. It is therefore necessary to look for a more sensitive approach to study it. Cardiotoxicity of aconite showed in the electrocardiogram is premature ventricular contractions (PVC), ventricular tachycardia, ventricular fibrillation, ventricular flutter until death according to the different poisoning degree (Sheikh-Zade et al., 2000; Wada et al., 2005). We think that measuring the minimum toxic dose for first PVC is a feasible way, which also provides a new idea for subsequent verification test. Last but not least, liquid phase conditions provided in this manuscript is a versatile method both for fingerprint identification and multi-component determination, which has a long analysis method and is not conducive to rapid testing. Currently, there were many literatures about the DDAs contents determination (Jiang et al., 2005; Kang et al., 2010), especially some UPLC methods, the analysis time was less than 25 min (Zheng et al., 2014). When determining the *TCI*, we can refer to the literatures to increase analysis rate.

It is the coming of Precision Medical era to promote us to consider how to achieve precise quality control and precise application of traditional herbal medicines. Aiming at the deficiency of multi-components determination, we first proposed the idea of active calibrated determination contents. Represented by the hypertoxic herb aconite, this manuscript demonstrated the study method of *TCI* and its accuracy and universality for quality control. We believe that this method is an accurate, quick and economic approach for toxicity evaluation of aconite without animal consumption, and it will offer a reference for other foods and herbs.

## Author Contributions

DZ, MY, JW, and XX contributed to the experimental design. DZ, RL, XH, and CL contributed to the animal experiments. DZ, XH, HZ, and ZZ contributed to the chemical analysis. DZ, RL, and JW contributed to data analysis. DZ and XH contributed to figure design and manuscript writing.

## Conflict of Interest Statement

The authors declare that the research was conducted in the absence of any commercial or financial relationships that could be construed as a potential conflict of interest.

## Abbreviations

AC: aconitine
Ch.P.: Chinese Pharmacopoeia
DDAs: diester diterpenoid alkaloids
HA: hypaconitine
HPLC: high performance liquid chromatography
MA: mesaconitine
MLD: minimum lethal dose
PLS-DA: partial least squares-discriminant analysis
PVC: premature ventricular contractions
RCT: randomized controlled trial
*TCI*: toxic constituents index
*TP*: toxic potency
UPLC: ultra-performance liquid chromatography
VF: ventricular fibrillation
VFL: ventricular flutter
VT: ventricular tachycardia.
